# Prevalence and factors associated with alcohol consumption among persons with diabetes in Kampala, Uganda: a cross sectional study

**DOI:** 10.1186/s12889-021-10761-5

**Published:** 2021-04-14

**Authors:** Maki Sifa Salama, Jonh Bosco Isunju, Salama Kaishusha David, Fiston Muneza, Sylvester Ssemanda, Nazarius Mbona Tumwesigye

**Affiliations:** 1 Makerere University, Kampala, Uganda; 2 Mulago Hospital, Kampala, Uganda; 3 St Francis Hospital Nsambya, Kampala, Uganda

**Keywords:** Persons with diabetes, Self-care behaviours, Alcohol consumption

## Abstract

**Background:**

The prevalence of diabetes has been rising increasing rapidly in middle- and low-income countries. In Africa, the World Health Organization projections anticipate diabetes mellitus to be the seventh leading cause of death in by 2030. Alcohol consumption influences diabetes evolution, in such a way that it can interfere with self-care behaviours which are important determinants of diabetes prognosis. In this study, we evaluated factors associated with alcohol consumption among persons with diabetes in Kampala to inform management policies and improve comprehensive diabetes care.

**Methodology:**

A cross-sectional study was conducted systematically among 290 adults with diabetes, attending diabetic clinics at Mulago National Referral Hospital and St Francis Hospital Nsambya. Data were entered and analysed in Epi-Info version 7 and STATA 13 software. Modified Poisson regression was used to identify factors associated with alcohol consumption among persons with diabetes. All tests were two-sided and the significance level for all analyses was set to *p* < 0.05.

**Results:**

The prevalence of alcohol consumption among persons with diabetes was 23.45% [95% CI: 18.9–28.7%]. Divorced, separated and widowed patients (Adj PR: 0.42, 95% CI: 0.21–0.83); and Protestant (Adj PR: 0.44, 95% CI: 0.24–0.82); Muslim (Adj PR: 0.30, 95% CI: 0.14–0.62); and Pentecostal (Adj PR: 0.32, 95% CI: 0.15–0.65) patients were less likely to consume alcohol. Diabetic patients who had a diabetes duration greater than 5 years were more likely to consume alcohol (Adj PR: 1.90, 95% CI: 1.25–2.88).

**Conclusion:**

Approximately one-quarter of participants consumed alcohol. However being catholic, never being married and having diabetes for more than 5 years predisposed persons with diabetes to alcohol consumption. Sensitization messages regarding alcohol consumption among persons with diabetes should be target patients who have never been married and those who have spent more than 5 years with diabetes; religion should also be considered as an important venue for health education in the community.

## Background

Diabetes mellitus is a global public health concern, with a steadily increase incidence [1, 2]. In 2012, an estimated 1.5 million deaths were directly caused by diabetes mellitus. The prevalence of diabetes has been increasing more rapidly in middle- and low-income countries [3]. In Africa, 12.1 million people were estimated to be living with diabetes in 2010, and this figure is projected to increase to 23.9 million people by 2030 [4]. According to the International Diabetes Federation, in Uganda there were 400,600 cases of diabetes in 2015 compared to approximately 98,000 in 2000 [5].

Alcohol consumption is detrimental among persons with diabetes and influences diabetes evolution [6]. It can also interfere with self-care behaviours, which are important determinants of diabetes prognosis because they are necessary for maintaining a good glycaemic control [6]. Addiction to alcohol among diabetic patients has been found to increase the risk of hyperglycemia, hypoglycemia, dehydration, high blood pressure, eye disease, damage to nerves, injuries and death [7–10].

While, diabetes is expected to increase by more than fifteen fold in Uganda over the next decade (Businge, 2010), the prevalence of alcohol consumption remains high [11] despite health education, and the existence of alcohol consumption guidelines and legislation. Up to 26.8% of the individuals in Uganda are current alcohol users, and the highest prevalence was found among people living in urban areas and in Central and Western regions, including Kampala [12, 13].

Despite the statistics from the Uganda Demographic and Health Survey (UDHS) that indicate an increase in diabetes incidence as well as an increase in alcohol consumption, information related to alcohol consumption among diabetic patients in Kampala is scant yet it is a public health problem that can be overcome through durable, persistent strategies. Previous literature on alcohol consumption in Uganda has focused mainly on people living with HIV [14], psychiatric patients [15] and the general population [16].

This study fills an information gap on both alcohol consumption and diabetes by determining the prevalence and identifying factors related to alcohol consumption among persons with diabetes. We will therefore increase the knowledge about alcohol consumption, especially that among diabetic patients. We will inform management policies, and we will guide the formation of evidence-based health promotion guidelines and strategies for secondary prevention, which are necessary to slow the development of diabetes complications and improve comprehensive diabetes care.

## Methods

### Study setting and design

A facility-based cross-sectional study was conducted between May and June 2017 among outpatients with diabetes, attending the two selected main hospitals (Mulago National Referral Hospital and St. Francis Hospital Nsambya) in Kampala.

Kampala is a Uganda city, with an estimated population of 1,659,600 habitants in 2011 according to the Uganda Bureau of Statistics (UBS).

The two main hospitals were selected purposively due to the larger number of people with diabetes enrolled in their diabetes clinics; they are teaching hospitals that treat patients from different districts, and with different socioeconomic levels. Mulago National Referral Hospital (MNRH)/Kirudu directorate (threating more than 100 patients/week) is a public hospital yet St Francis (treating almost 30 patients/week) is a private not-for-profit hospital run by the Uganda Catholic Medical Bureau Kampala. Both health facilities have good record management, have diabetic outpatient clinics that open once a week and almost all stable diabetic patients are given a medical appointment every 2 months.

### Study population

The study population was composed of people with diabetes, who visited St. Francis Hospital, Nsambya and MNRH diabetic clinic; during the study period. The study included all persons with diabetes aged eighteen and above, who were followed up for at least 1 year, at the diabetic clinics of St. Francis Hospital Nsambya and MNRH during the time of the study. Very sick patients who were unable to respond to the questionnaire and patients who did not sign the consent form were excluded.

### Sample size determination

The sample size was estimated using the formula for cross-sectional studies developed by Kish Leslie [17] as follows: $n=\left[\frac{Z^2 PQ}{\delta^2}\right]$ with 95% confidence interval (Z = 1.96), assuming that 45% of persons with diabetes consume alcohol (from Saint Francis Nsambya Hospital record, 2016), and assuming an absolute difference (d) of 0.06 and a 10% nonresponse rate. A total sample size of 290 persons with diabetes was required.

The diabetic patients who were followed at each hospital each week on the day of the diabetes clinic composed the study sample. Therefore 68 patients (15 patients/week) were selected at St Francis Hospital and 222 (50 patients/week) were selected at Mulago. Data were collected at St Francis Hospital Nsambya and at MNRH, on each clinic day, and the systematic sampling approach was used to enrol patients. During the seven-week study period, the sampling interval was two in each hospital. This means that every second patient was chosen, until the desired sample size was achieved.

### Data collection methods

Data were collected using a pretested interviewer-administered questionnaire in English and in Luganda (the main Ugandan local language).

During patients medical care visit at the diabetes clinic, the study aim was explained to those who were systematically selected and met the eligibility criteria. All diabetic patients who agreed to participate in the study provided a signed informed consent form in accordance with the Makerere University Faculty of Medicine Research and Ethics Committee (FOMREC) guidelines.

The data collection tools were anonymous and included a structured questionnaire that was completed by research assistants; who had good knowledge of diabetes and who were trained before the commencement of the study in accordance with the study aim, data collection methods and research ethics.

The study involved only a quantitative data collection method, which was used to calculate percentages and test the relationships between variables.

The questionnaire included sociodemographic characteristics of the participants (age, gender, marital status, religion, tribe, occupation, residence, and others), clinical data about diabetes (type of diabetes, time spent with diabetes, health education about diabetes, and others), level of knowledge on alcohol consumption and diabetes (signs of alcoholism, effect of alcohol on the body, effect of alcohol on diabetes and others), and personal information about alcohol consumption within the last year.

The level of knowledge on diabetes control and alcohol consumption was assessed on the basis of the adapted Substance Use Knowledge, Attitude and Practice Survey questionnaire [18]. Regarding the main signs of alcoholism [19], three questions were asked, and the participants who responded properly to 2 questions were considered to have a good level of knowledge on signs of alcoholism. There were nine questions about the effects of alcohol on the body and the participants who respond properly to five questions were considered to have good knowledge on the effects of alcohol consumption on the body. Questions on the effects of alcohol consumption on diabetes were asked, and the participants who responded properly to four questions of the eight questions asked were considered to have good knowledge of the effects of alcohol consumption on diabetes.

The health education variable assessed whether patients received health education at the facility during the routine visit (yes or no), who delivered the message, and at what frequency (never, always, and sometimes).

Information on alcohol consumption within the last year, was self-reported by the patients; and then categorized into binary outcome variables (yes or no). For those who reported consuming alcohol, we further classified their alcohol consumption into 5 categories; using the Alcohol Use Disorders Identification Test (AUDIT) questionnaire [20, 21]: non- drinkers; any alcohol drinking; alcohol misuse (scoring 3 to 7 points for men or 3 to 8 points for women points or higher on the AUDIT tool); hazardous alcohol drinking (scoring 8 or more points for men and 7 or more points for women on the AUDIT tool) and binge alcohol drinking (corresponding to 6 or more drinks on a single occasion for men or 5 or more drinks on a single occasion for women at least once last year). In this study, nondrinkers and any alcohol drinkers (both having an AUDIT score less than three) were put in the same categories of “nondrinkers” as they carry low risks in developing complications [20–22].

The reasons for alcohol consumption were reported by the patient were categorized into family influence, pleasure or peer influence and means of coping with stress (health worries, work stressors, etc.). Additionally, each patient reported the type of alcohol that was usually taken (beer, wine, and spirit/local brews).

### Data analysis

Data were field edited, coded, cleared and checked for consistency. Coding was performed to clearly identify the required variables for analysis. The data were entered into Epi-Info version 7, transferred to Microsoft Excel 13 for cleaning, and then exported to STATA 13 software for statistical analyses. Summary statistics including frequencies and proportions for categorical variables were performed, and means with their standard deviations (SDs) were obtained for continuous variables. We identified factors associated with alcohol consumption (included only alcohol misuse, hazardous alcohol drinking and binge drinking) among persons with diabetes, by using both bivariate (to check for associations and relationships between alcohol consumption and the predictors) and modified Poisson regression analysis (Poisson regression with robust error variance) to obtain estimates that are relatively robust to omitted covariates, as the prevalence of alcohol consumption among persons with diabetes was greater than 10% [23].

Variables that were significant in the bivariate analysis were included in modified Poisson regression model, and the inclusion criterion was *p* ≤ 0.05. The forward elimination method was then used to build the statistical model and hence to determine factors that were associated with alcohol consumption among persons with diabetes. All statistical tests were two sided. To measure the strength of association, we used the prevalence ratio (PR). We reported crude and adjusted prevalence ratios with their 95% confidence intervals and *p* values. The significance level for all the analyses was set to *p* ≤ 0.05.

## Results

### Sociodemographic characteristics of participants

This study was performed at MNRH/Kirudu Directorate and at St Francis Hospital Nsambya in Kampala and data were collected between May and June 2017.

From Table 1, the mean age (SD) of the respondents was 51.4 (±14.8) years. Approximately 42.4% (123/290) of the study participants were in the age range of 31 to 50 years followed by 37.5% (109/290) in the age range of 51 to 70 years. The majority of the study participants were female (52.4%, 152/290) and approximately 64.8% (216/290) of the participants were married. Most of the study participants resided in Makindye division (48.2%, 140/290), followed by those residing in the Kawempe division 28.2% (82/290).

**Table 1:** Sociodemographic and clinical characteristics of the study participants

Characteristics	Univariate Analysis		
	Men ***n*** = 138 n(%)	Women ***n*** = 152 n (%)	Total ***n*** = 290 n (%)
Age (years)			
18–30	14 (10.1)	10 (6.5)	24 (8.2)
31–50	64 (46.3)	59 (38.8)	123 (42.4)
51–70	48 (34.7)	61 (40.1)	109 (37.5)
> 70	12 (8.6)	22 (14.4)	34 (11.7)
Marital Status			
Never married	20 (14.5)	10 (6.5)	30 (10.4)
Married	107 (77.5)	81 (53.3)	188 (64.8)
Divorced/Separated/Widowed	11 (8)	61 (40.2)	72 (24.8)
Level of education			
No education	19 (13.7)	46 (30.2)	65 (22.4)
Primary	49 (35.5)	51 (33.5)	100 (34.4)
Secondary	50 (36.2)	44 (28.9)	94 (32.4)
Tertiary	20 (14.4)	11 (7.2)	31 (10.6)
Residence (Division)			
Central	10 (7.2)	21 (13.8)	31 (10.6)
Makindye	72 (52.1)	68 (44.7)	140 (48.2)
Kawempe	46 (33.3)	36 (23.6)	82 (28.2)
Nakawa and Rubaga	6 (4.3)	14 (9.2)	20 (6.8)
Other districts	4 (2.8)	13 (8.5)	17 (5.8)
Religion			
Catholic	58 (42)	64 (42.1)	122 (42)
Protestant/Anglican	29 (21)	31 (20.3)	60 (20.6)
Muslim	28 (20.2)	35 (23)	63 (21.7)
Pentecostal & Others	23 (16.6)	22 (14.4)	45 (15.5)
Tribe			
Baganda	94 (68.1)	101 (66.4)	195 (67.2)
Basoga	9 (6.5)	14 (9.2)	23 (7.9)
Banyankore/Bakiga	17 (12.3)	23 (15.1)	40 (13.7)
Others	18 (13)	14 (9.2)	32 (11)
Occupation			
Not working	13((9.4)	65 (42.7)	78 (26)
Salary earner	38 (27.5)	24 (15.7)	62 (21.3)
Farmer	14 (10.1)	17 (11.1)	31 (10.6)
Self employed	73 (52.8)	46 (30.2)	119 (41)
Hospital			
Private	29 (21)	39 (25.6)	68 (23.4)
Public	109 (78.9)	113 (74.3)	222 (76.6)
Type of Diabetes			
Type 1	12 (8.6)	3 (1.9)	15 (5.2)
Type 2	126 (91.4)	149 (98)	275 (94.8)
Times with Diabetes			
1 year - < 5 years	53 (38.4)	69 (45.3)	122 (42)
5 years - < 10 years	46 (33.3)	25 (16.4)	71 (24.4)
10 years- < 15 years	16 (11.5)	31 (20.3)	47 (16.2)
≥ 15 years	23 (16.6)	27 (17.7)	50 (17.2)
Health education			
Never	98 (71)	102 (67.1)	200 (68.9)
Always	6 (4.3)	5 (3.2)	11 (3.7)
Sometimes	34 (24.6)	45 (29.6)	79 (27.2)
Good Knowledge on alcoholism and Diabetes:			
-Do not have	12 (8.6)	12 (7.8)	24 (8.2)
-Sign of alcoholism	41 (29.7)	47 (30.9)	88 (30.3)
-Sign of alcoholism + effects of alcohol on the body	67 (48.5)	78 (51.3)	145 (50)
-Sign of alcoholism + effects of alcohol on the body+ effects of alcohol on diabetes	18 (13)	15 (9.8)	33 (11.3)

The majority of diabetic patients were self-employed (41%, 119/290) and approximately 34.4% (100/290) of the study participants attained a primary level of education, whereas 32.4% (94/290) attained a secondary level education. Regarding tribe and religion, the majority of the participants (67.2%, 195/290) were Baganda and Catholic (42%, 122/290) respectively (Table 1).

The results in Table 1, show that approximately three-quarters of the participants were from the public hospital 76.6% (222/290). Among persons with diabetes, 94.8% (275/290) had type 2 diabetes, yet the proportion of patients with type 1 diabetes was only 5.2% (15/290), and approximately 42% (122/290) had spent between one and 5 years with diabetes (Table 1).

Most of the participants did not receive health education regarding diabetes when they came for routine visits at the hospital 68.9% (200/290). (Table 1).

As shown in Table 1, most of the participants did not receive health education regarding diabetes when they came for routine visits at the hospital 68.9% (200/290), and the majority of study participants (50%, 145/290) had good knowledge of signs of alcoholism and the effects of alcohol on the body, followed by those with of signs of alcoholism (30.3%, 88/290).

### Alcohol consumption among patients with diabetes

**Fig. 1:**
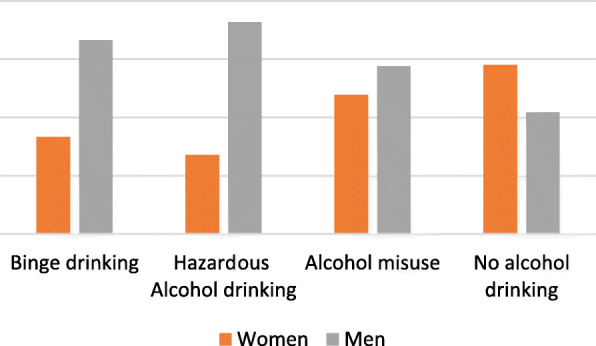
Prevalence and categories of alcohol consumption among persons with Diabetes

Based on the AUDIT questionnaire and alcohol consumption self-report, 23.45% (68/290) [95% CI: 18.9–28.7%] of persons with diabetes who visited the MNRH and St Francis Hospital Nsambya, consumed alcohol, and the majority were men (66.17%, 45/68) [95% CI: 54.06–76.48%] as shown in Fig. 1.

Most of the participants, consumed alcohol hazardously (11.3%, 33/290) [95% CI: 8.1–15.6%], followed by those who misused it (8.9%, 26/290) [95% CI: 6.1–12.8%] and binge drinking was reported by 3.1% (9/290) [95% CI: 1.6–5.8%] of the study participants (see Fig. 1).

**Fig. 2:**
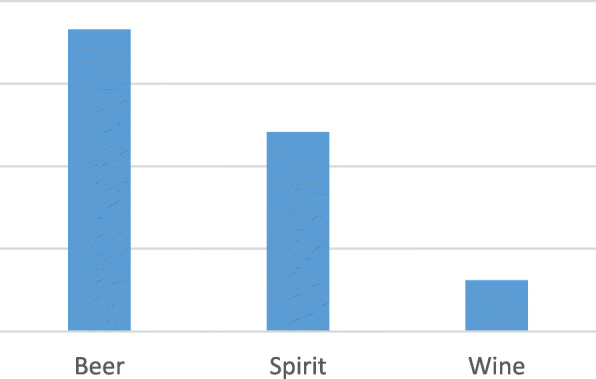
Type of alcohol consumption, by sex

**Fig. 3:**
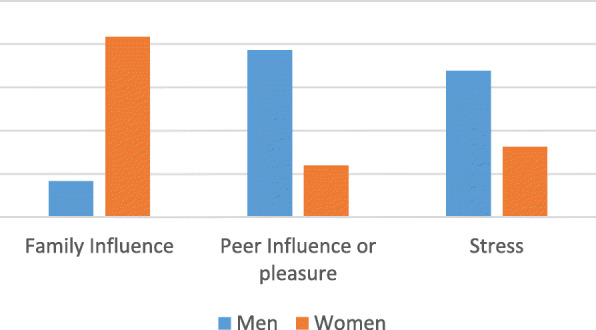
Reasons of alcohol consumption among diabetic patients, by sex

The majority of the persons with diabetes consumed beer (77.9%, 53/68) as shown in Fig. 2; and 58.8% (40/68) of patients reported means of coping stress as a major reason of alcohol consumption (see Fig. 3).

### Factors associated with alcohol consumption among persons with diabetes

Multivariable Poison regression models were used to analyse factors that were related to alcohol consumption among persons with diabetes followed at MNRH and at Saint Francis Hospital Nsambya.

The variables revealed as independent predictors for alcohol consumption among persons with diabetes in Kampala were religion, marital status and time spent with the disease.

The prevalence of alcohol consumption among Pentecostal/Protestant/ Anglican followers was 68 and 56% lower, respectively, than that among Catholic followers, and the difference was statistically significant (Adj PR: 0.32, 95% CI: 0.15–0.65; Adj PR: 0.44 95% CI: 0.24–0.82). In addition, Muslims were less likely to consume alcohol than Catholics, which was statistically significant (Adj PR: 0.30, 95% CI: 0.14–0.62) (Table 2).

**Table 2:** Factors associated with alcohol consumption (bivariate and multivariable analysis) among persons with Diabetes

Characteristics	Alcohol consumption		Bivariate Analysis		Multivariate Analysis	
	No	Yes	UPR (95% CI)	***P*** Value	Adj PR (95% CI)	***P*** Value
Sex						
Male	93	45	1.0		1.0	
Female	129	23	**0.46 (0.29–0.72)**	0.001	0.74 (0.48–1.14)	
Age (years)						
18–30	16	8	1.0			
31–50	87	36	0.87 (0.46–1.64)			
51–70	89	20	0.55 (0.27–1.09)			
> 70	30	4	0.35 (0.11–1.04)			
Marital Status						
Never married	19	11	1.0		1.0	
Married	140	48	0.69 (0.40–1.18)	0.181	0.80 (0.51–1.25)	0.345
Divorced/Separate/Widowed	63	9	**0.34 (0.15–0.73)**	0.006	**0.42 (0.21–0.83)**	0.013
Level of education						
No education	55	10	1.0			
Primary	76	24	1.56 (0.79–3.04)			
Secondary	69	25	1.72 (0.89–3.35)			
Tertiary	22	9	1.88 (0.85–4.17)			
Residence (Division)						
Central	27	4	1.0			
Makindye	100	40	2.21 (0.85–5.74)			
Kawempe	62	20	1.89 (0.79–5.09)			
Nakawa and Rubaga	17	3	1.16 (0.28–4.66)			
Other districts	17	1	0.45 (0.55–3.77)			
Religion						
Catholic	76	46	1.0		1.0	
Protestant/Anglican	51	9	**0.39 (0.20–0.75)**	0.005	**0.44 (0.24–0.82)**	0.009
Muslim	56	7	**0.29 (0.14–0.61)**	0.001	**0.30 (0.14–0.62)**	0.001
Pentecostal/Others	39	6	**0.35 (0.16–0.77)**	0.009	**0.32 (0.15–0.65)**	0.002
Tribe						
Baganda	147	48	1.0			
Basoga	18	5	0.88 (0.39–1.99)			
Banyankore/Bakiga	31	9	0.91 (0.48–1.71)			
Others	26	6	0.76 (0.35–1.63)			
Occupation						
Not working	68	10	1.0		1.0	
Salary earner	47	15	1.88 (0.91–3.91)	0.088	1.35 (0.63–2.81)	
Farmer	25	6	1.50 (0.59–3.80)	0.382	1.27 (0.51–3.16)	
Self employed	82	37	**2.42 (1.28–4.59)**	0.007	1.67 (0.86–3.26)	
Hospital						
Private	55	13	1.0			
Public	167	55	1.29 (0.75–2.22)			
Type of Diabetes						
Type 1	12	3	1.0			
Type 2	210	65	1.18 (0.41–3.30)			
Times with Diabetes						
1 year - < 5 years	97	25	1.0		1.0	
5 years - < 10 years	42	29	**1.99 (1.27–3.12)**	0.003	**1.90 (1.25–2.88)**	0.002
10 years- < 15 years	43	4	0.41 (0.15–1.13)	0.086	0.59 (0.22–1.55)	0.288
≥ 15 years	40	10	0.97 (0.50–1.88)	0.942	1.09 (0.58–2.04)	0.787
Health education						
Never	152	48	1.0			
Always	6	5	1.89 (0.94–3.79)			
Sometimes	64	15	0.79 (0.47–1.32)			
Good Knowledge on alcoholism and Diabetes:						
-Do not have	152	48	1.0			
-Sign of alcoholism	55	17	0.89 (0.43–1.83)			
-Sign of alcoholism + effects of alcohol on the body	3	1	0.80 (0.40–1.60)			
-Sign of alcoholism + effects of alcohol on the body+ effects of alcohol on diabetes	12	2	0.41 (0.13–1.26)			

Patients who had spent 5 to 10 years with diabetes were more likely to consume alcohol than those who had spent less than 5 years with diabetes. (Adj PR: 1.90, 95% CI: 1.25–2.88) (Table 2).

The prevalence of alcohol consumption among divorced, separated and widowed patients was 58% lower than that among patients who had never been married. (Adj PR: 0.42, 95% CI: 0.21–0.83) (Table 2).

## Discussion

Alcohol consumption remains a long-standing public health issue in Uganda. Alcohol consumption can be harmful to vulnerable people with diabetes, by interfering with self-care behaviours and affecting important organs in the body. Therefore, this study fills a knowledge gap by detecting factors associated with alcohol consumption among persons with diabetes in Kampala, to improve comprehensive diabetes care by providing possible strategies and interventions and informing management policies.

One-quarter of diabetic patients treated in the two selected health facilities (MNRH and St Francis Hospital) in Kampala consumed alcohol. Alcohol use in Uganda is widely accepted in local culture and tradition. Additionally, Uganda is abundantly supplied with alcoholic beverages (beer, wine, liquor produced in factories in the country or imported and informally produced beer and distilled liquor in local makeshift bars and homes), such as Heineken, Tusker, Guinness, Bell, Nile Special and Club. The findings were similar to a countrywide estimate of the prevalence of alcohol use in Uganda that showed an overall prevalence of current alcohol use of 26.8% [12]. However, the prevalence of alcohol consumption in this study was much lower than that in a study conducted among individuals with type 2 diabetes from 20 different countries in the world, where up to 30% of patients were found to drink alcohol [22], and another study conducted in northern California among adults with diabetes revealed a prevalence of 50% [6]. That prevalence was much higher than the one revealed in a study conducted among Croatians (5.8%) [24]. This must be due to differences in sociodemographic and cultural characteristics among the different study populations.

In this study, the majority of people with diabetes, consumed alcohol hazardously. Additionally among those who consumed alcohol hazardously and who reported binge drinking, the main reason for their drinking was stress. In addition to life events that are inherently stressful, diabetic patients also have to overcome the stress of their disease. In the present era of modernization, balancing work, family, leisure time and a chronic disease along with all its requirements is a big challenge for patients, and may increase their stress level. Studies have revealed that alcohol consumption is strongly associated with stress. Alcohol consumption is mostly used as a means of coping with stress [25, 26]. Chronic stress can therefore interfere with a diabetic patient’s capacity to adhere to self-care behaviours which are essential for maintaining good health [27]. Our findings emphasize the importance of regular screening for stress as a component of routine diabetes care to identify and manage stress early. This will help to improve Glycemic control as well as quality of life and prognosis.

The majority of persons with diabetes consume beer, followed by spirits and wine. This is different from studies done in Uganda among HIV patients and studies done in the USA and Croatia, where the majority of patients consume wine, followed by beer and spirits [6, 14, 24]. Uganda is abundantly supplied with alcoholic beverages which mostly include beers such as Tusker, Guinness, Bell, Nile, Eagle and Club. Those beverages are cheaper, they are always available in retail and local shops, and they can also be consumed in public places and even at home. Guidelines regulating alcohol production and commercial sales, time and place restrictions for selling alcohol, density of outlets and advertisements practices must be studied further.

Religion was significantly associated with alcohol consumption. Catholics were more likely to consume alcohol; because in the Catholic religion, alcohol consumption is not prohibited, contrary to other religions, which consider alcoholic beverages to be incompatible with a holy life, so abstaining from alcohol is an obligation in those religions. This result is similar to other studies performed in Uganda [14, 16] and other countries [6, 28, 29] where Catholic followers were more likely to consume alcohol than followers of other religions. According to the WHO, religion might play a role in the prevention of alcohol consumption [30]. Therefore, religion can be used strategically to reduce alcohol-related problems among persons with diabetes. By providing health education to followers, the information can be disseminated throughout the populations.

The duration of the disease was significantly associated with alcohol consumption. This was consistent with other studies performed in Asia and Africa where patients with a diabetes duration of ≤5 years were more adherent to diet, especially regarding alcohol intake, than those who had a duration of > 5 years [31–34].

According to Glasgow et al., the duration of disease appears to have a negative relationship with diet adherence [35, 36]. In 2010 Egede and Ellis showed that despondency can also be a factor influencing poor dietary practice regarding alcohol consumption among diabetic patients [37].

In most health facilities in Uganda, patients presenting with diabetes are initially encouraged to maintain a diet that includes avoiding alcohol consumption, to prevent complications. Over time, health education can be neglected due to lack of motivation, lack of time, absence of family and health care support, and patients becoming fed up with following a dietary regimen. In that sense, health professionals need to double their attention to newly and formerly diagnosed diabetic patients, to provide them solid support in terms of health education. They need to discuss in detail the importance of self-care behaviours that include avoiding alcohol, because the reason for discontinuing such behaviours after 5 years of the disease duration could be inadequate diabetic education or consultation and a decrease in motivation over time.

This study shows that never married diabetic patients consume more alcohol than widowed patients. This is similar to a study conducted in the USA in 2016 in the general population where never married people were more likely to consume alcohol than married and widowed people [38]. This finding is also similar to a study performed among women in Accra (Ghana) [39]. Widows have more responsibilities than never married people, especially in regard to taking care of children. Therefore, instead of purchasing alcohol, they tend to use the majority of their resources for their children’s needs. Additionally, they have to spend less time with friends and coworkers and more time with their children, which may reduce alcohol consumption.

### Study limitations and strengths

Recall bias could have occurred as some data, especially from the questionnaire, were self-reported by the person with diabetes. The other limitation in this study is a social desirability bias that could have occurred since most of the information was reported by participants. Persons with diabetes who also drink alcohol may not disclose fully to the interviewers the extent of their drinking.

The strengths of this study include the use of the AUDIT questionnaire, a standardized internationally-validated tool for alcohol assessment in primary care settings, allowing for cross-study comparability. Furthermore, previous studies were focused on the general population or on specific groups, such as HIV and psychiatric patients. This study examined alcohol consumption among persons with diabetes in Kampala, where there is a continuous increase in diabetes incidence.

## Conclusion and recommendations

Approximately one-quarter of persons with diabetes treated at MNRH and St Francis Hospital outpatient diabetes clinic, in Kampala consume alcohol. Being widowed, Protestant, Muslim or Pentecostal, and having spent less than 5 years with diabetes were associated with lower alcohol consumption.

Religion is an important venue for health education against alcohol-related problems among persons with diabetes. The sensitization message regarding alcohol consumption among persons with diabetes should be target mainly never married people and those who have spent more than 5 years with the disease. Further study must be done to identify the temporal relationship between time spent with diabetes and alcohol consumption among diabetic patients.

## Data Availability

The dataset used and analysed during this study is available from the corresponding author.

## Abbreviations

AUDIT: Alcohol Use Disorder Identification Test
AUD: Alcohol Use Disorder
MNRH: Mulago National Referral Hospital
UDHS: Uganda Demographic and Health Survey
WHO: World Health Organization
UPR: Unadjusted Prevalence Ratio
Adj PR: Adjusted Prevalence Ratio
CI: Confidence Interval
